# Association between air pollution in Lima and the high incidence of COVID-19: findings from a post hoc analysis

**DOI:** 10.21203/rs.3.rs-39404/v2

**Published:** 2021-03-15

**Authors:** Bertha V. Vasquez-Apestegui, Enrique Parras-Garrido, Vilma Tapia, Valeria M. Paz-Aparicio, Jhojan P. Rojas, Odón R. Sánchez-Ccoyllo, Gustavo F. Gonzales

**Affiliations:** Cayetano Heredia University; Cayetano Heredia University; Cayetano Heredia University; Cayetano Heredia University; Servicio Nacional de Meteorología e Hidrología del Perú; Universidad Nacional Tecnólogica de Lima Sur; Cayetano Heredia University

**Keywords:** Air pollution, social distancing, particulate matter, long-term exposure, fatality rate

## Abstract

**Background:**

Corona virus disease (COVID-19) originated in China in December 2019. Thereafter, a global logarithmic expansion of the cases has occurred. Some countries have a higher rate of infections despite of early implementation of quarantine. Air pollution could be related to the high susceptibility to SARS-CoV-2 and the associated case-fatality rates (deaths/cases*100). Lima, Peru has the second highest incidence of COVID-19 in Latin America, and it is also one of the cities with highest levels of air pollution in the Region.

**Methods:**

This study investigated the association of the levels of PM_2.5_ exposure in the previous years (2010–2016) in 24 districts of Lima with the positive-cases, deaths and case-fatality rates of COVID-19. Multiple Linear regression was used to evaluate this association controlled by age, sex, population density and number of food markets per district. The study period was from March 6 to June 12, 2020.

**Results:**

There were in Lima 128,700 SARS-CoV-2 positive cases, and 2,382 deaths due to COVID-19. The case-fatality rate was 1.93%. Previous exposure to PM_2.5_ (years 2010–2016) was associated with number of Covid-19 positive-cases (*β* = 0.07; 95% CI: 0.034–0.107) and deaths (*β* = 0.0014; 95% CI: 0.0006–0.0.0023), but not with case-fatality rate.

**Conclusions:**

the higher rates of COVID-19 in Metropolitan Lima is attributable, among others, to the increased PM_2.5_ exposure in the previous years after adjusting for age, sex and number of food markets. Reduction of air pollution since a long-term perspective, and social distancing are needed to prevent spreads of virus outbreak.

## Background

Corona virus disease (COVID-19) is caused by the severe acute respiratory syndrome corona virus 2 (SARS-CoV-2) and was first reported in December 2019 from Wuhan in the Hubei province of the Republic of China [1]. On March 11, 2020, due to the global logarithmic expansion of the cases, COVID-19 was declared a pandemic by the World Health Organization (WHO) [2]. On March 6, 2020, the Peruvian president announced the first COVID-19 infection case in Lima – an imported case of a Peruvian national who had returned from recent travel to France, Spain, and the Czech Republic [3]. On March 11, 2020, the President declared a general quarantine with social distancing interventions, including closures of all educational institutions (i.e., schools and universities) in Peru, and on March 16, 2020, a national emergency was declared [3].

Three months after the declaration of the emergency and strict measures of social isolation, the rates of infection were extremely high in the metropolitan area of Lima than in the rest of the country. As of June 12, 2020, there were 128,700 (58.3%) COVID-19 cases in Lima and 92,049 (41.7%) in the rest of the country. Several reasons have been suggested to explain the high incidence of COVID-19 and the inability to reduce the scaling up of the COVID-19 outbreak.

A similar situation with a high incidence of the outbreak in one specific region of the country that was different that in other regions has been described in Italy. In this country, the highest incidence of COVID-19 has been observed in the northern part of the country [4; 5].

Several studies have explored the association between SARS-CoV-2 transmission and the COVID-19 mortality rate with environmental factors, air pollution being among them [6; 7].

One explanation of the higher rate of SARS-CoV-2 infection is that the susceptibility of the population to the virus is predetermined by the exposure to air pollutants in the previous years [6]. A higher rate of infection with COVID-19 in northern Italy was apparently related to the highest levels of air pollution in that part of the country [8]. It is suspected that individuals with chronic PM_2.5_ exposure suffer progressive and chronic inflammation of the respiratory tract and are more prone to severe respiratory diseases after viral infections [4]. Epidemiological evidence associates chronic PM_2.5_ exposure to health problems [9; 10; 11]. Nonetheless, in a recent study, researchers did not observed association between PM_2.5_ exposures with COVID-19 transmission during the first months of the pandemic [12].

Lima is one of the most polluted cities in Latin America [13], and it is possible that long-term exposure to air pollutants may increase the infection susceptibility of individuals to different external agents, including bacteria and viruses [14; 15; 16]. In the city of Lima, PM_2.5_ concentrations vary by seasonality based on meteorological conditions. In late autumn and winter (lower temperatures), the gas–particle conversion processes increase the environmental PM_2.5_ concentrations [17]. Another possibility is that the virus could be loaded onto the particulate matter (PM), which then acts as a vector for infection spread, extending the persistence of the viral particles in the air, thereby favoring an “indirect” transmission in addition to the direct spread (individual to individual) [5].

Based on these arguments, it has been suggested that a reduction in the air pollution that occurred secondary to the shutting down of national and international transportation could reduce the disease spread [18; 19] and thus may explain the decreased incidence of COVID-19.

The experience in Peru seems to demonstrate that the reduction in air pollution was unassociated with reduction of COVID-19 cases. The daily PM_2.5_ concentrations showed a gradual decrease from March 16 and did not exceed the environmental quality standards for air that were specified by the Peruvian Ministry of the Environment. On average, for Lima, a 38% decrease for PM_2.5_ was recorded (during the first 15 days of the state of emergency) compared to its historical concentrations (2015–2019) [20]. However, in the same period the cases of COVID-19 in Lima (1338 × 100,000 inhabitants) increased much more than in the rest of the country (399 × 100,000 inhabitants), representing 58% of the cases from all the country.

Unlike other governments worldwide, the Government of Peru declared quarantine as soon as the first case was detected. After the declaration of the emergency, the level of air pollutants was greatly reduced although new cases of COVID-19 continued increasing.

We hypothesized that air pollution could have a chronic effect that increases the susceptibility of individuals to the virus and that individuals living in places with high air pollution in the years preceding the pandemic are at a higher risk of infection.

To evaluate this hypothesis, this study involved the analysis of data from 24 districts of Lima that were characterized by different levels of PM_2.5_ exposure in the years preceding the COVID-19 outbreak to evaluate whether the values obtained in previous years were associated with the incidence and mortality rates of COVID-19.

The primary objective was to determine if long-term exposure with different PM_2.5_ concentrations was associated with the number of cases, death and case-fatality rates to COVID-19. Then, we determined if this association is modified by age, sex, and number of food markets per district.

## Methods

This research was undertaken as an ecological study that involved the analysis of two secondary databases. We assessed the data of COVID-19 cases and deaths that occurred in Lima metropolitan area until June 12, 2020. We comparatively analyzed the data on COVID-19 with the estimate daily levels of PM_2.5_ measured in the years between 2012 and 2016 [21],

The district was taken as the unit of analysis. In this study, 24 districts of Lima were included: Ate, Barranco, Carabayllo, Chorrillos, Comas, El Agustino, Independencia, La Molina, La Victoria, Lima, Lince, Los Olivos, Puente Piedra, Rímac, San Borja, San Isidro, San Juan de Lurigancho, San Juan de Miraflores, San Luis, San Martín de Porres, Santiago de Surco, Surquillo, Villa el Salvador, and Villa María del Triunfo. These districts included a total population of 7,029,238 inhabitants and a population density of 241,623 people per square kilometer.

PM_2.5_ data were obtained from the National Meteorology and Hydrology Service of Peru (SENAMHI). SENAMHI has ten stations that records PM_2.5_ daily concentrations in Lima. Data was obtained as a part of an agreement between SENAMHI and Universidad Peruana Cayetano Heredia as part of the Regional GEOHealth Hub centered in Peru. Details of the construction of database were published previously [21]. Other pollutants were not included because there was insufficient district-level data for the analysis. Data on the population and surface areas of the provinces and altitude of the capitals of the provinces in Peru were obtained from the Peruvian Center for planning website [22]

In the 24 districts, there were 94,273 COVID-19 cases and 1,987 deaths. The case-fatality rate by COVID-19 was 2.58% in Peru and 1.93% in Lima. In the 24 districts of Lima (Peru), the number of food markets was 948. The data from the COVID-19 database of the Open Data website of Peru [23] was used to analyze the information on COVID-19 deaths and SARS-CoV-2-positive individuals.

### Statistical analysis

Data were managed in the MS Excel 2016 program. The STATA v14.0 statistical package (StataCorp, College Station, TX, USA) was used for analysis and ArcGis 10.5 was used for maps construction. First, the average amounts of PM_2.5_ were calculated by each district from 2012 to 2016 (Supplementary table 2). Data on the age at infection and at death are presented as mean ± standard deviation, and the differences between the pair of means were calculated by the Student’s *t*-test. The number of COVID-19 cases and deaths by each district was evaluated overall and by each sex.

We explored distinct linear models to assess the relationship of the reported positive cases and deaths, by sex and the sex ratio, at the district level of the province of Lima.

As secondary objective, we assessed the association between number of food markets per district and cases and deaths due to COVID-19. Data from the number of food markets in each district were obtained from a survey in 2016 [24].

The associations between the cases, deaths due to COVID-19 and case-fatality rate (deaths/cases of COVID-19*100) and previous PM_2.5_ exposure were evaluated by using linear regression controlled by age, sex, population density and number of food markets per district. Statistical significance was considered at *p* < 0.05.

## Results

By June 12, 2020, Peru had 220,749 SARS-CoV-2 positive cases and 6,308 deaths. Lima had 128,700 SARS-CoV-2 positive cases and 2,382 deaths due to COVID-19. Among all the identified positive cases and COVID-19 deaths, 59.1% (N = 130,462) and 71.1% (N = 4,485), respectively, were men. The number of deaths in the 24 districts studied represents 94.8% of all COVID-19 deaths in Lima.

The national COVID-19 case-fatality rate was 2.58% and was 1.93% for Lima. The mean age at infection was 20 years lower than the age at death due to COVID-19. The patterns at the country and Lima Metropolitan area were similar. Women were infected and succumbed at later ages than men (Table 1).

**Figure 1:**
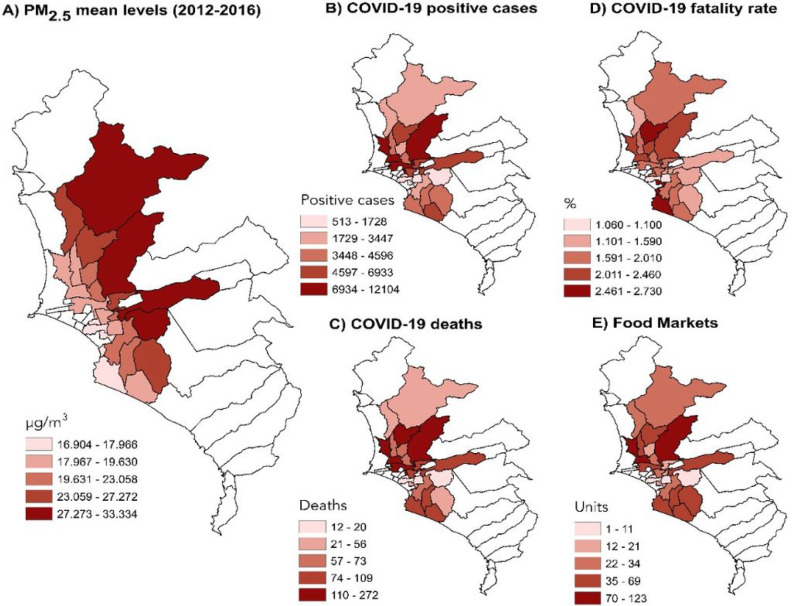
The distribution of air pollution and COVID-19 cases in Lima. A) particulate matter ≤2.5 μm (PM2.5), B) Incidence of COVID-19 cases, C) Incidence of COVID-19 deaths, D) COVID-19 fatality rate (Deaths/Cases*100), and E) abundance of food markets. Environmental data are expressed as μm/m3 and refers to the mean values for 2012–2016. The distribution data for COVID-19 were obtained from the Ministry of Health of Peru (COVID-19 data updated until June 12, 2020).

The mean concentrations of PM_2.5_ from 2012 to 2016 for the 24 districts evaluated in this study are shown in Fig. 1A. The lighter red zone refers to lower levels of PM and the darkest tone refers to the highest level of this pollutant.

The incidence of COVID-19 cases and deaths are shown in Fig. 1B and 1C, respectively. The highest incidence of cases and deaths occurred in the districts located to the north of the city compared with the other zones evaluated. The COVID-19 case-fatality rates (Deaths/Cases*100) in Lima are shown in Fig. 1D, and the abundance of food markets per district is shown in Fig. 1E. Moreover, the largest numbers of markets were present in the districts with the highest COVID-19 incidence of cases and deaths (north of the city).

**Figure 2:**
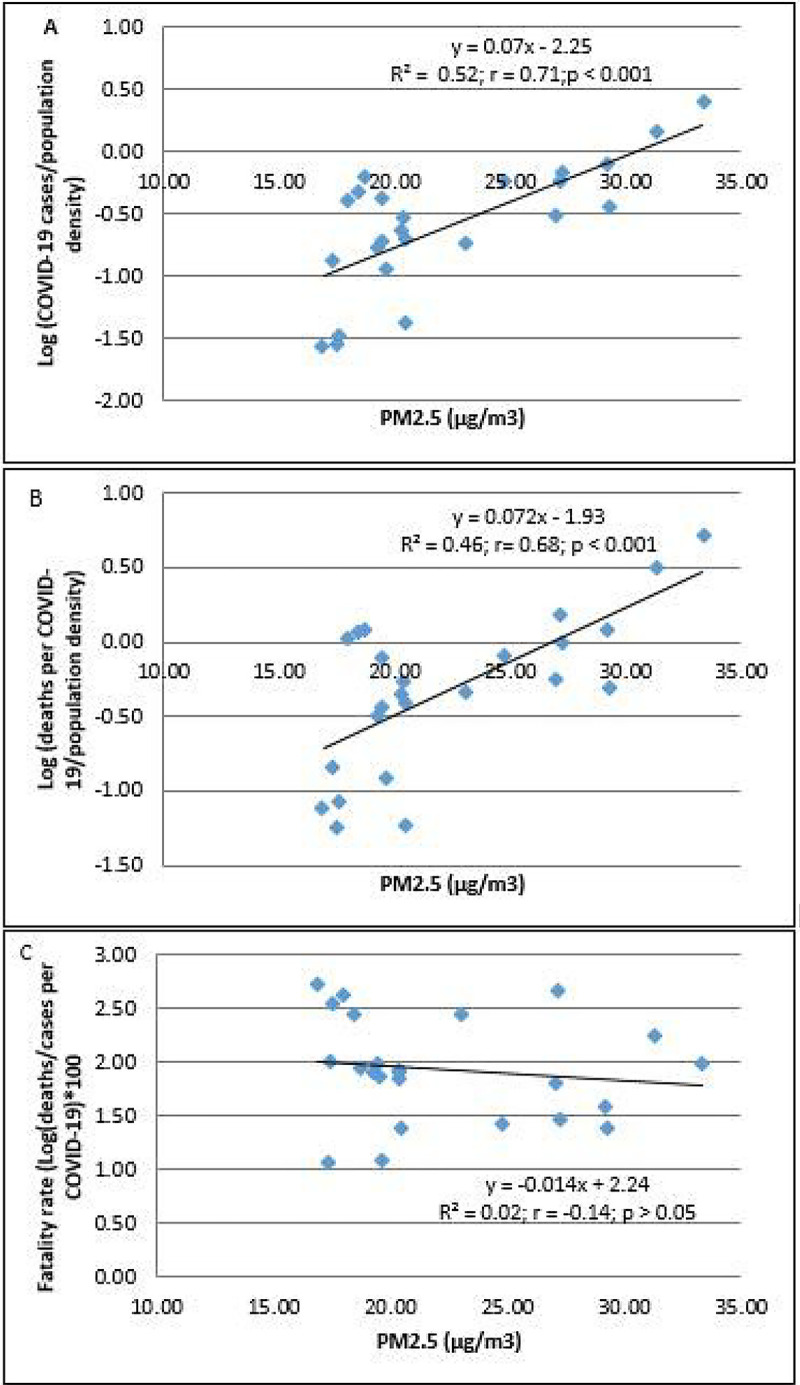
The association between PM2.5 and Log (cases of COVID-19/population density) (A), Log (deaths per COVID-19/population density) (B), and fatality rates *100 (C) in 24 districts of Metropolitan Lima. The population of the district was ascertained from the values reported in the census of 2017.This number was not corrected based on estimation of growth.

Higher PM_2.5_ levels were associated with the higher incidence of COVID-19 cases and deaths in 24 districts of Metropolitan Lima (Figs. 2A and 2B). However, the case-fatality rate did not increase with the increasing levels of PM_2.5_ (Fig. 2C).

**Figure 3:**
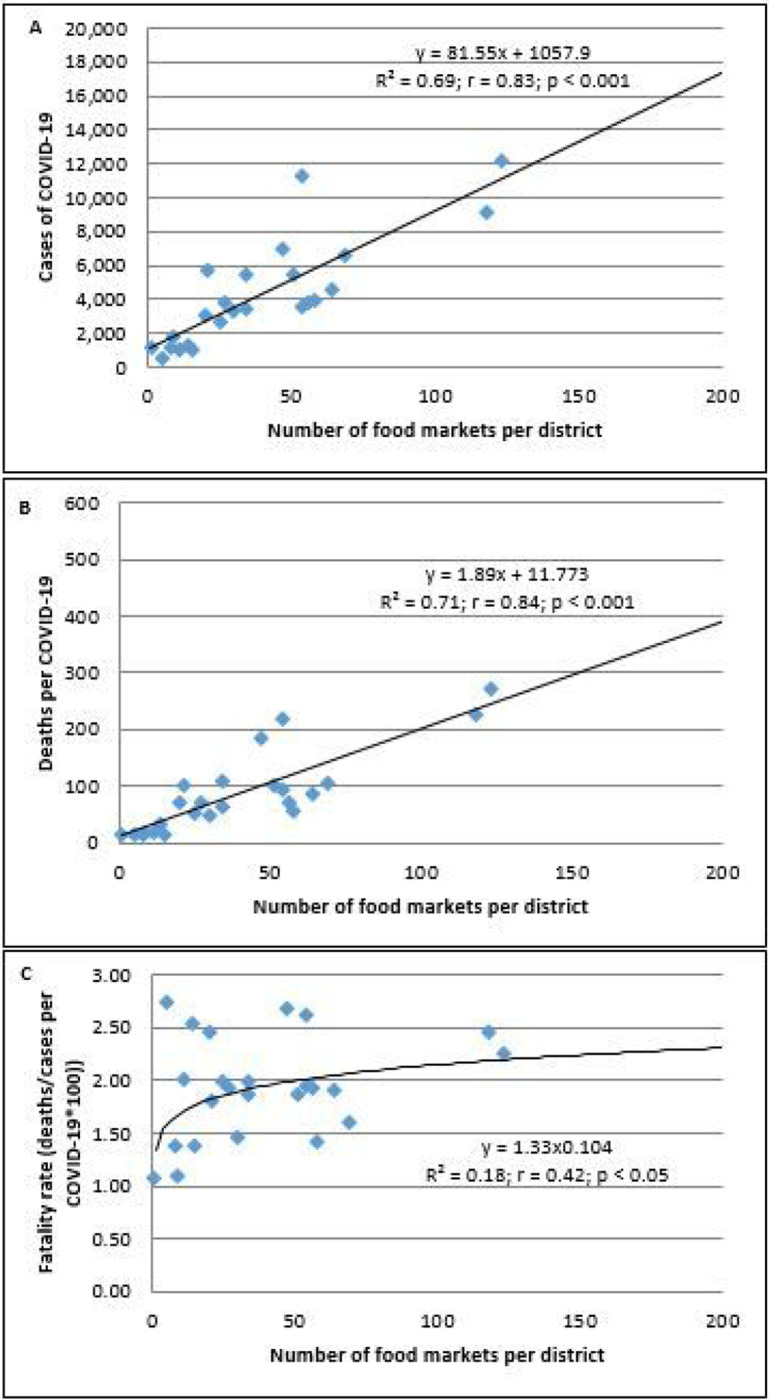
The association between the number of food markets per district and Log (cases of COVID-19/population density) (A), Log (deaths per COVID-19/population density) (B), and fatality rates *100 (C) in 24 districts of Metropolitan Lima.

Similarly, a higher number of food markets were linearly associated with the higher number of cases and mortality of COVID-19 (*p* < 0.01, Fig. 3A and *p* < 0.01, Fig. 3B, respectively).

The association between the number of food markets with the cases and mortality of COVID-19 persisted when cases (R^2^ = 0.25; r= 0.49; *p* < 0.01) and deaths (R^2^ = 0.33; r= 0.58; *p* < 0.01) were adjusted by population density. Furthermore, the case-fatality rate was associated with the increased number of food markets (*p* < 0.05; Fig. 3C).

The number of food markets per district did not correlate with PM_2.5_ (μg/m^3^) (Y = 0.033x + 21.05; R^2^ = 0.04; r = 0.21; *p* > 0.05). This suggests that PM_2.5_ and the number of food markets were independently associated with the spread of COVID-19; moreover, the higher number of food markets was associated with an increased case-fatality rate whereas the higher PM_2.5_ level did not increase the fatality rate (data not shown).

Furthermore, the higher the number of food markets per district, the higher was the incidence of COVID-19/population density (y = 0.03x^063^; r = 0.58; *p* < 0.01). The higher the number of food markets per district, the higher was the COVID-19 mortality/population density*100 (Y = 0.04x ^073^; r = 0.66; *p* < 0.001; data not shown).

**Figure 4:**
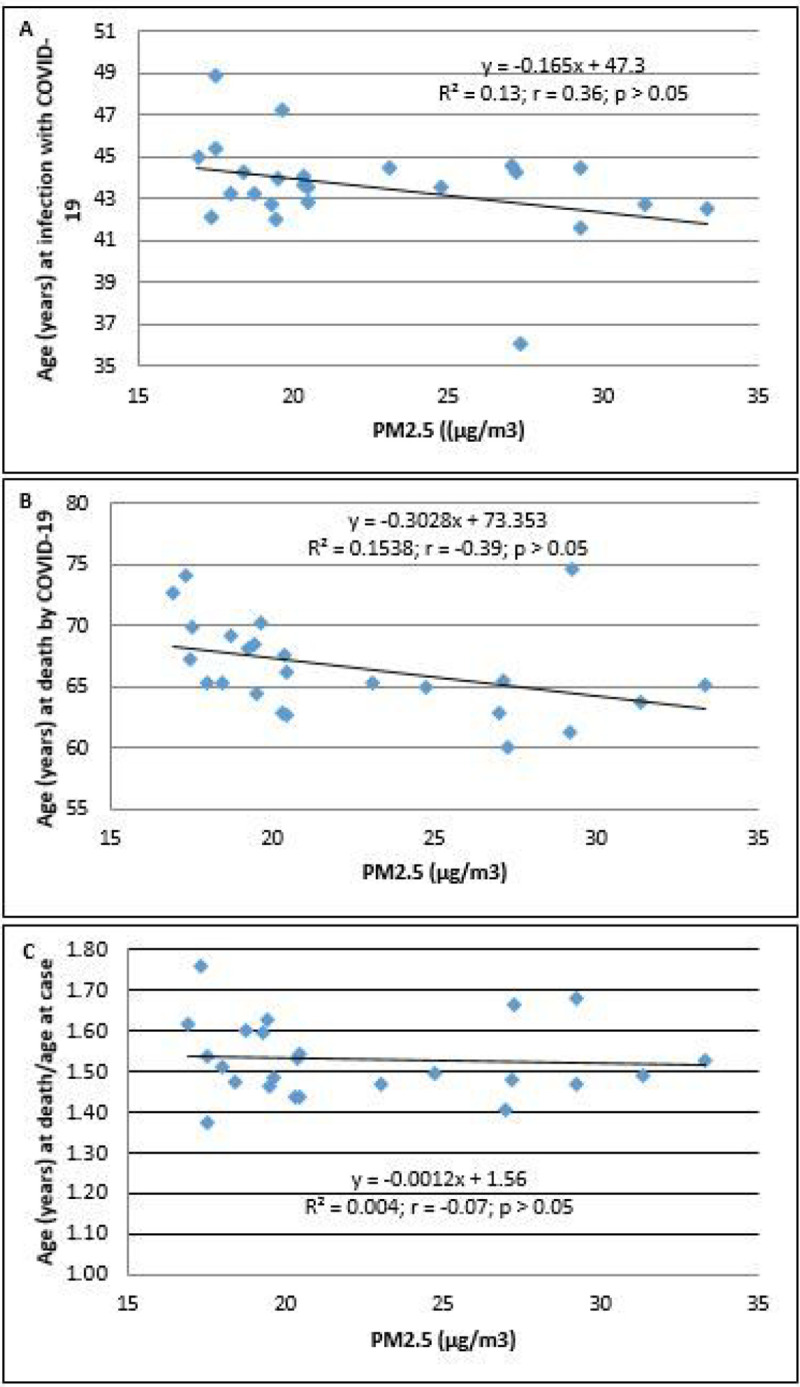
The association between PM2.5 (μg/m3) and age (years). (A) Age at SARS-CoV-2 infection, (B) age at death due to COVID-19, and (C) age at death/age at confirmation of COVID-19 in 24 districts of Metropolitan Lima. The population of the district was ascertained from the values reported in the census of 2017.This number was not corrected based on estimation of growth.

The increasing PM_2.5_ levels were not associated with specific age at infection, deaths, or the age at death/age at infection for COVID-19 (*p* > 0.05; Figs. 4A, 4B, and 4C).

The GLM analysis showed that the increasing levels of PM_2.5_ (μg/m^3^) in the previous years (2012–2016) was associated with the higher incidence of COVID-19 in the 24 districts of Lima that were included in the study. This was observed after adjusting the sex ratio, age at onset, and the number of food markets per district (Table 2). Table 3 shows that PM_2.5_ was associated with deaths/population density after controlling age, sex and number of food markets. The case-fatality rate (deaths/cases*100), after controlling for different variables was not associated regarding the increasing levels of PM_2.5_ (Table 4).

## Discussion

This study showed that exposure to high levels of PM_2.5_ in the years preceding the COVID-19 pandemic was associated with the higher incidence of SARS-CoV-2 infection and mortality due to COVID-19 but without affecting the case-fatality rate (Deaths/cases*100) in Metropolitan Lima, a city that is considered one of the most polluted in Latin America [13]. This suggests that the current incidence of COVID-19 is associated with a chronic exposure to air pollution. The association was maintained including after controlling by population density, age, sex and number of food markets.

This is an important finding that explain why cases of COVID-19 increased in Lima despite Peru had one of the earliest COVID-19 lockdowns in Latin America applying a quarantine, with much of the activities closed (schools, universities, churches, ban of public events) soon after the first case of COVID-19 was detected.

A previous systematic analysis showed that the incidence of and the risk of morbidity and mortality from COVID-19 increase with chronic and acute exposure to air pollution, particularly to PM (PM_2.5_ and PM_10_) and nitrogen dioxide [8; 25; 26; 27].

The values of PM_2.5_ ranged for all districts from 14.50 to 41.69 μg/m^3^, all of which were higher than the annual mean value declared by the WHO (10 μg/m^3^). Thus, as it was previously shown that PM_2.5_ was associated with an increasing risk for respiratory infectious diseases [16], the present situation with the COVID-19 spread demonstrates an increasing trend. We have assessed the association with long-term PM_2.5_ exposure; however, the impact of short-term exposure needs to be addressed in further research.

An analysis of the distribution of COVID-19 cases worldwide demonstrates a remarkable asymmetry with respect to the countries/regions [28; 29]. This is exactly the pattern that we have observed in Metropolitan Lima. From the 24 districts assessed, those with higher PM_2.5_ concentrations during 2012–2016 showed more COVID-19 incidence than did those with less concentrations of the pollutants (Supplementary Table 2). Nationwide, 220749 SARS-CoV-2 positive cases and 6,308 deaths due to COVID-19 have been reported since the first case was reported. Lima has 58.3% of the national cases and 38% of deaths. However, Lima represents only 28% of the total Peruvian population.

Human pathogenic corona viruses, which include SARS-CoV-2 that is responsible for COVID-19, bind to their target cells through the angiotensin-converting enzyme 2 (ACE2) receptor expressed by epithelial cells of the lung, intestine, kidney, and blood vessels [30]. It is possible that PM_2.5_ may induce the elevation of ACE2 activity.

Active cigarette smoking up-regulates ACE-2 expression in the lower airways, which may partially explain the increased risk of severe COVID-19 in these populations [31]. If air pollutants act similarly as the agents released during smoking, it is probable that individuals with chronic exposure to these compounds would have low ACE-2 activity or greater susceptibility to the infection [32].

This study in Metropolitan Lima that used data of PM_2.5_ from 2012 and 2016 for 24 districts showed that the case-fatality rate did not increase with the increasing values of PM_2.5_. This is an interesting finding because a crude analysis of the data showed that deaths are higher when PM_2.5_ concentrations are higher. However, when the data are calculated as the amount of deaths/number of cases due to COVID-19 (case-fatality rate), there was no observable association with PM_2.5_. However, the results suggest that PM_2.5_ does not affect the case-fatality rate. This agrees with the data observed in New York after short-term exposure to air pollutants [6].

The national COVID-19 case-fatality rate was 2.58%, and 1.93% for Lima. The highest rate in the rest of Peru respect to Lima may reflect the deficiencies in the healthcare system in the different provinces of Peru. The COVID-19 case-fatality rate was higher in men and increased with age, thus confirming previous results [33].

In average, the age at infection was around 42 years in Peru. After end of quarantine, it is possible that age at infection will lowered. This due to that more of young people will be exposed.

At national and at province level (Lima), the age at infection was 20 years lower than the age at death due to COVID-19. This higher mortality risk for older people has been reported previously [34; 35]. In our study, the association between older age and COVID-19 mortality risk was unaffected by increasing levels of PM_2.5_, suggesting that the factors that explain the higher mortality risk with age are independent of PM_2.5_ exposure. In fact, PM_2.5_ did not modify the COVID-19 case-fatality rate in Lima.

Lima has an average of 13% elderly population and 22% under 19 years of age. The districts of the suburbs (Ate, Puente Piedra, Carabayllo, VES and VMT) have the lowest elderly population and therefore more young people. The largest population (19–64 years) is similar in all districts, and those who move around the city for work, shopping, entertainment, are the most exposed to infection [36].

There is evidence of the role of PM pollutants in SARS-CoV-2 transmission. PM_2.5_ and other small PM can act as disease vectors and facilitate the airborne transmission of viable virus particles, and these have been incriminated in the spread of measles and SARS [37].

Social distancing is an important preventable measure to decrease the spread of COVID-19. This was demonstrated in 28 European countries, where the most probable point of change during the COVID-19 epidemic showed a dose-response association of the observed flattening of the epidemic curve with an increasing social distancing index (SDI). Countries in the highest SDI quartile achieved a statistically significant decline in the incidence and prevalence of the epidemic [37].

In Brazil, according to a recent report, social distancing measures that were adopted by the population appeared effective, particularly when implemented in conjunction with the isolation of cases and quarantining of contacts [38].

In numerical simulations, in a city within Brazil, three scenarios were compared: first was the vertical distancing policy, where only older people was distanced; the second involved the horizontal distancing policy where all age groups adhered to social distancing; and the third involved a control scenario wherein no intervention was undertaken to distance people. Horizontal distancing, if applied with the same intensity in all age groups, significantly reduced the total number of infected people by “flattening the disease growth curve”; however, vertical distancing or non-distancing did not show this effect [39].

Doubtless, social distancing measures appear to be the most effective intervention to slow the disease spread of COVID-19. Though studies unanimously confirm the mitigating effect of social distancing on disease spread, the reported effectiveness varies from 10% to a more than 90% reduction in the number of infections [37]. The changes of mobility in public places, such as retail and recreation centers (e.g., restaurants, cafes, theaters, etc.), grocery stores and pharmacies, transit hubs (e.g., airports, bus stations, subways, etc.), and parks, are the most important determinants of the disease-transmission rate [40].

In Peru, some specific factors could contribute to the spread of COVID-19 during quarantine. These include the easy availability of food markets, banks, and public transport. In Peru, the food markets remained open during the quarantine period to ensure food availability for the population.

According to the findings of this study, in districts where there were more markets, there were higher numbers of cases and deaths. This may explain the high spread of the COVID-19 cases in Lima, in a situation in which individuals are susceptible to the virus by previous exposure to air pollutants.

It is well known that the highest risk of SARS-CoV-2 transmission occurs prior to symptom onset. A recent paper provides evidence of the effectiveness of mask use, disinfection, and social distancing in the prevention of COVID-19 [41].

In this study, the number of markets was unrelated to the PM_2.5_ concentration, which suggests that both factors are independently associated with the spread of COVID-19. Moreover, after controlling for different variables including number of food markers, PM_2.5_ remains associated to the number of cases of COVID-19.

From a long-term perspective, the reduction of air contamination should be considered a part of the integrated approach for sustainable development, human health protection, and for reducing the *spread* of a disease during *an outbreak, epidemic,* or *pandemic.* However, although reducing air pollution is important to reduce morbidity and mortality due to different diseases, the findings of this study suggest also the importance of social isolation to reduce the incidence of COVID-19 [42]. The magnitude of contagion in the food markets is an example that policies that are aimed at reducing crowding could be important for preventing the spread of COVID-19.

The limitations of the study are lack of data on number of people attending the food markets during the quarantine period. We were also unable to obtain data on people attending banks, and public transport within the same period. Data also on accomplish of social distancing and use of mask is also lacking.

The study makes a significant contribution to the literature because the findings indicate that higher PM_2.5_ levels are associated with higher incidence of infections with SARS-CoV-2 and mortality of COVID-19. However, the case-fatality rate did not increase with the increase in PM_2.5_ levels.

The findings of this study are generalizable to regions with similar population density and PM_2.5_ levels in the setting of respiratory epidemics or future pandemics. This study will be a support tool for decision-making in the country’s health policy, since having a study in which PM_2.5_ is associated by district, age, sex, markets of stocks in the number of cases of COVID-19 and deceased, will allow to rethink the measures used by the Peruvian government, and other countries characterized by high air pollution.

In conclusion, the present study demonstrated that the higher rates of spread of COVID-19 in Metropolitan Lima (Peru) were associated to the previous long-term PM_2.5_ exposure. Men and older people were at higher risk of death due to COVID-19. Reduction of air pollution since a long-term perspective, and social distancing are needed to prevent spreads of virus outbreak. These results must be considered by officers of the Governments to be applied in the health policies aimed to prevent or reduce epidemic viral spread. The strategies taken to confront the pandemic must consider previous environmental indicators to intensify efforts in areas with higher air pollution.

## Figures and Tables

**Table 1:** Sex-stratified differences in the age at COVID-19 confirmation and at death due to COVID-19 at the national level and in the province of Lima

Statistical variables	Age among men (years)	Age among women (years)

Cases at country level	42.93 ±16.88	43.17 ±1 7.69*
	(n = 130,333)	(n = 90,256)

Cases at Lima level	43.01 ±16.90	43.67 ±17.96*
	(n = 72,992)	(n = 50,613)

Deaths at country level	64.51 ±13.85	66.68 ±14.91*
	(n = 4,047)	(n = 1,643)

Deaths at Lima level	64.43 ±14.12	67.37 ±14.47*
	(n = 1695)	(n = 687)

Data are mean ± SD.

* *p* < 0.01, with regard to the values in men.

**Table 2:** The association between the COVID-19 cases/population density and previous PM_2.5_ concentrations in the 24 districts of Lima

COVID-19 case/population density	Crude coefficient	95% CI		Adjusted coefficient	95% CI	
PM_2.5_	0.083**	0.050	0.115	0.070**	0.034	0.107
Sex ratio	−3.133*	−5.329	−0.937	−2.157	−5.127	0.812
Age	−0.080	−0.181	0.021	0.047	−0.064	0.158
Food markets	1.269	−0.259	2.796	0.242	−0.973	1.457

* *p* < 0.05

** *p* < 0.01.

The model was adjusted for the PM_2.5_ level, sex ratio (female cases: male cases), food markets (number of food markets per district and age [years] at the COVID-19 diagnosis). Data for the PM_2.5_ level (μg/m^3^) correspond to the average data per district obtained daily from 2012 to 2016.

**Table 3:** The association between the COVID-19 deaths/population density and previous PM_2.5_ concentrations in the 24 districts of Lima

COVID-19 deaths/population density	Crude coefficient	95% CI		Adjusted coefficient	95% CI	
PM_2.5_	0.0016**	0.0008	0.0023	0.0014*	0.0006	0.0023
Sex ratio	0.019	−0.005	0.044	0.009	−0.010	0.029
Age	−0.001	−0.002	0.000	0.000	−0.001	0.002
Food markets	0.029	−0.002	0.060	0.024	−0.007	0.055

* *p* < 0.05

** *p* < 0.01.

The model was adjusted for the PM_2.5_ level, sex ratio (female deaths: male deaths), food markets (number of food markets per district and age [years] at the moments of deaths by COVID-19). Data for the PM_2.5_ level (μg/m^3^) correspond to the average data per district obtained daily from 2012 to 2016.

**Table 4:** Association between the COVID-19 case-fatality rate and previous PM_2.5_ concentrations in the 24 districts of Lima

COVID-19 fatality rate	Crude coefficient	95% CI		Adjusted coefficient	95% CI	
PM_2.5_	−0.014	−0.056	0.029	0.01	−0.047	0.068
Sex ratio	−0.303	−2.569	1.963	−1.956	−5.207	1.293
Age	0.043	−0.049	0.135	0.035	−0.039	0.11
Food markets	1.082	−0.257	2.422	0.0054	−0.001	0.012
Population density	0.00001	−6.21e-^06^	.000035	0.00003	−2.60e-^06^	0.00006

*p* > 0.05.

The model was adjusted for PM_2.5_ level, sex ratio (female cases: male cases), age [years], food markets (number of food markets per district and population density at the COVID-19 diagnosis). Data for the PM_2.5_ level (μg/m^3^) correspond to the average data per district obtained daily from 2012 to 2016.

## Data Availability

The datasets generated and/or analyzed during the current study are available in the following repositories: • **Data of food markets:** INEI repository: www.inei.gob.pe/media/MenuRecursivo/publicaciones_digitales/Est/Lib1447/libro.pdf • **Data related to COVID-19: MINSA repository:** https://www.datosabiertos.gob.pe/dataset/casos-positivos-por-covid-19-ministerio-de-salud-minsa https://www.datosabiertos.gob.pe/dataset/fallecidos-por-covid-19-ministerio-de-salud-minsa • **Environmental data:** Data was obtained as a part of an agreement between SENAMHI and Universidad Peruana Cayetano Heredia as part of the Regional GEOHealth Hub centered in Peru. The datasets of predicted PM_2.5_ concentrations at 1 Km^2^ spatial resolution in Lima-Peru from 2010 to 2016 used during the study are available from the corresponding author on reasonable request.

## Abbreviations

ACE2: Angiotensin-converting enzyme 2
CI: Confidence interval
COVID-19: Disease caused by caused by the severe acute respiratory syndrome corona virus 2 (SARS-CoV-2)
°C: Centigrade degree
GEOHealth: Global Environmental and Occupational Health
GLM: Generalized Linear Model
Log: Logarithm with base 10
P: Probability
PM2.5: Fine particulate matter
PM10: Solid or liquid particles whose diameter varies between 2.5 and 10 μm
R2: Coefficient of determination
R: Coefficient of Pearson
SARS: Severe acute respiratory syndrome
SARS-CoV-2: Severe acute respiratory syndrome coronavirus 2
SD: Standard deviation
SDI: Social distancing index
SENAMHI: National Service of Meteorology and Hydrology of Peru
SIDISI: Decentralized Information System and Monitoring of Research
WHO: World Health Organization
